# A cross-sectional study exploring the relationship between the dietary inflammatory index and hyperlipidemia based on the National Health and Nutrition Examination Survey (2005–2018)

**DOI:** 10.1186/s12944-023-01908-x

**Published:** 2023-08-31

**Authors:** Yu Han, Xijuan Jiang, Yabin Qin, Yile Zhao, Guying Zhang, Chao Liu

**Affiliations:** 1 Department of Pharmacy, Hebei Children’s Hospital, Shijiazhuang, Hebei People’s Republic of China; 2 Department of Laboratory Animal Science, Hebei Medical University, Shijiazhuang, Hebei People’s Republic of China; 3 Hebei Key Lab of Laboratory Animal Science, Shijiazhuang, Hebei People’s Republic of China; 4 Department of Pharmacology, Hebei Medical University, Shijiazhuang, Hebei People’s Republic of China

**Keywords:** Diet, Inflammation, Dietary inflammatory index, Hyperlipidemia, National Health and Nutrition

## Abstract

**Background:**

Hyperlipidemia is closely associated with dietary patterns and inflammation. However, the relationship between hyperlipidemia and the inflammatory potential of diets remains unexplored. The research was conducted to examine the relationship between hyperlipidemia and dietary inflammatory index (DII).

**Methods:**

The data utilized in the research were acquired from the National Health and Nutrition Examination Survey (NHANES) from 2005 to 2018. The information on dietary intake was gathered by conducting 24-h dietary recall interviews. Restricted cubic spline (RCS) and Survey-weighted logistic regression were utilized to determine the association between DII and hyperlipidemia. Furthermore, stratification analysis was carried out.

**Results:**

This study included 8982 individuals with and 3458 without hyperlipidemia. Participants with hyperlipidemia exhibited higher DII scores than those without hyperlipidemia. Following adjustment for gender, age, race, education level, marital status, poverty, drinking status, diabetes, hypertension, smoking status, body mass index (BMI), chronic kidney disease (CKD), cardiovascular disease (CVD), and hemoglobin (Hb), the association between the prevalence of hyperlipidemia and DII remained significant. The RCS data demonstrated that the hyperlipidemia prevalence did not exhibit an increase until the DII score was approximately 2.78. Stratification analysis revealed that the association between DII and hyperlipidemia persisted in all subgroups.

**Conclusions:**

DII was associated with hyperlipidemia, and the threshold DII score for the risk of hyperlipidemia was 2.78.

## Background

Hyperlipidemia is a prevalent medical condition defined by increased lipid levels in the blood, especially cholesterol and triglycerides levels [1]. The condition is widely prevalent, particularly in developed countries. According to recent studies, the prevalence of hyperlipidemia varies across different populations [2, 3]. Hyperlipidemia is linked to multiple health conditions, such as cardiovascular diseases (CVD), which remain the leading cause of death [4].

Currently, some studies have investigated the lipid-lowering action of some foods or nutrients, such as dietary fibers and plant sterols [5, 6]. However, most studies only analyze certain nutrients and cannot provide a thorough understanding of the association between dietary patterns and hyperlipidemia, characterized as pro-inflammatory or anti-inflammatory diets. The Dietary Inflammation Index (DII) is an effective method for evaluating diet inflammatory potential [7]. Shivappa et al. reported the details of the development of DII. DII computes the inflammatory effects of diets based on 45 different nutrients, comprising pro- and anti-inflammatory nutrients [8]. A higher DII score indicates a greater inflammatory effect of the diet, whereas a lower DII score suggests that the diet is more likely to be anti-inflammatory. Elucidating the relationship between DII and hyperlipidemia could help in early interventions in hyperlipidemia patients by changing their dietary patterns.

The association between DII and hyperlipidemia has not been elucidated. This research intends to determine the relationship between hyperlipidemia and DII using the data obtained from the National Health and Nutrition Examination Survey (NHANES), which is a cross-sectional survey of the US civilian population.

## Materials and methods

### Study population and design

The data utilized in this study were accessed at the NHANES, which evaluates the nutritional status and health of the non-institutionalized civilian population. The NHANES utilizes a multistage, stratified, sophisticated, probability-sampling design for evaluating the prevalence of common health conditions and assessing the associated risk factors. The NHANES protocols were sanctioned by the National Center for Health Statistics Research Ethics Review Board, and informed consent was obtained from all the individuals before their inclusion in the surveys. The comprehensive details of the NHANES methods and procedures are provided at http://www.cdc.gov/nchs/nhanes.htm.

**Fig. 1:**
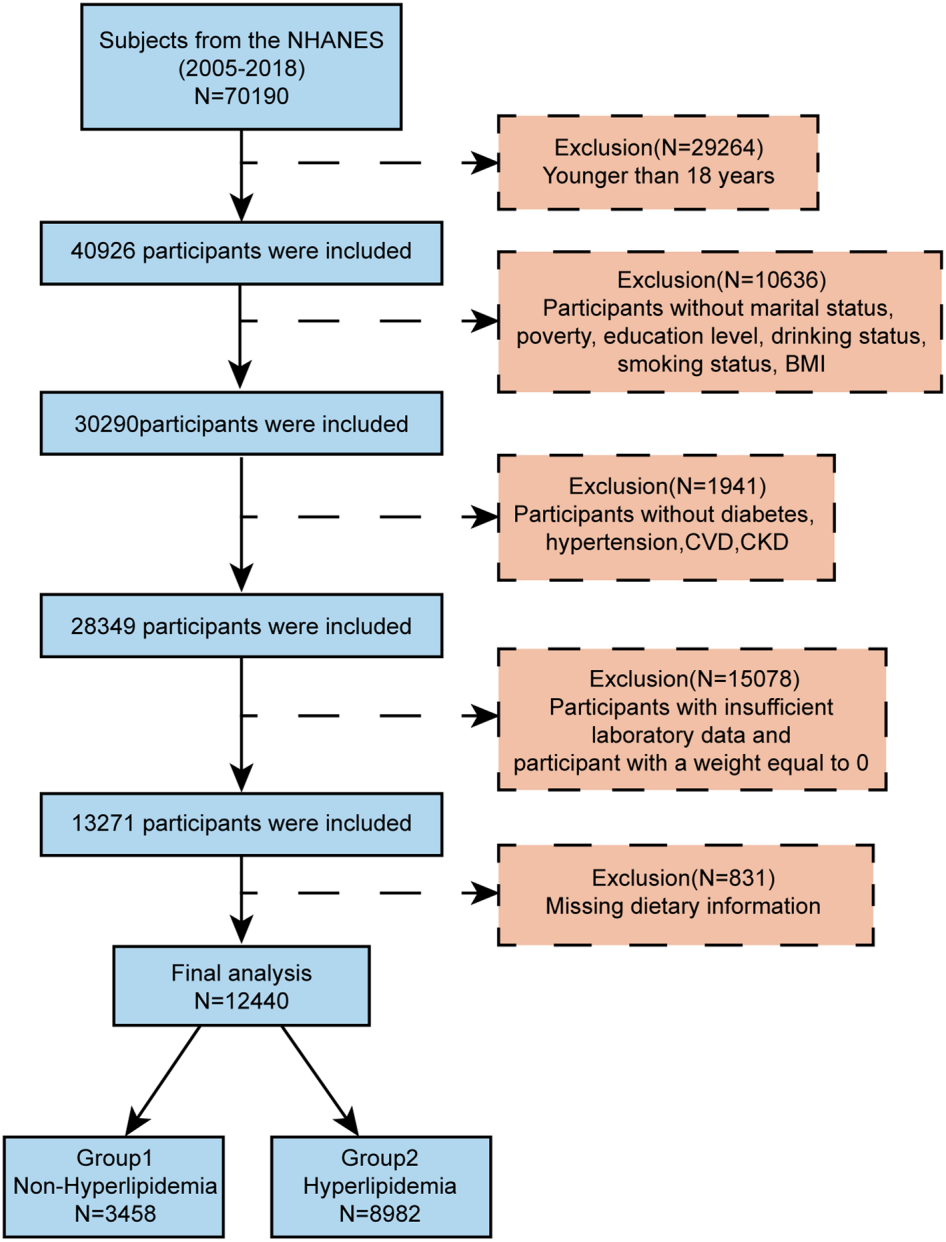
Flow chart of the study subjects

Data obtained from the NHANES 2005–2018 were combined to increase the sample size and decrease the sampling error. The analysis was restricted to participants aged ≥ 18 years. Participants who lacked dietary information and those with missing information on the covariables of interest were excluded. In total, 12,440 eligible participants from the NHANES were included in this study. In order to make a nationally representative estimate account for all statistical analyses, sampling weights were applied (Fig. 1).

### Exposure

DII was used to evaluate the diet inflammatory potential. When the number of nutrients used to calculate DII was < 30, the DII was still considered valid [8, 9]. This study used the following 28 food parameters to compute the DII: carbohydrates, total fat, protein, alcohol, saturated fat, fiber, cholesterol, polyunsaturated fatty acid (PUFA), monounsaturated fatty acids (MUFA), n-6 and n-3 fatty acids, as well as thiamin, riboflavin, niacin, vitamins (A, B6, B12, C, D, and E), iron, magnesium, zinc, folic acid, selenium, caffeine, beta-carotene, and energy [10]. To evaluate the DII, the Z-score was derived using the equation “(daily mean intake − global daily mean intake)/standard deviation” and then converted to a percentage score. To obtain symmetric distribution, the score was doubled for each percentile and subtracted from “1”. The food parameter-specific DII was obtained by multiplying the percentile of each food parameter with its respective overall inflammatory effect score. The details of the DII calculation was reported by Shivappa et al. [11]. In this study, DII was analyzed as a continuous variable, transformed from a continuous to a categorical variable, and divided into first tertile (T1), second tertile (T2), and third tertile (T3).

### Hyperlipidemia

The hyperlipidemia status of the participants was evaluated based on the National Cholesterol Education Program. Hyperlipidemia was defined as triglycerides ≥ 150 mg/dL, total cholesterol ≥ 200 mg/dL, low-density lipoprotein (LDL) ≥ 130 mg/dL, or high-density lipoprotein (HDL) ≤ 50 mg/dL in females and ≤ 40 mg/dL in males [12]. Moreover, participants who reported using lipid-lowering medications were considered to have hyperlipidemia [13, 14]. Hyperlipidemia was diagnosed if any of the five conditions was met.

### Covariables

Covariables contained gender, age, race, marital status, poverty, education level, drinking status, smoking status, body mass index (BMI), diabetes, hypertension, CVD, chronic kidney disease (CKD), hemoglobin (Hb), fasting glucose (glucose), and estimated glomerular filtration rate (eGFR). Among the covariables, race was categorized as black, white, Mexican, or other. The education levels were classified as college, high school, or less than high school (including the participants who studied up to or beyond college level). Poverty was categorized according to the family monthly poverty level index, with < 1.30 defined as low, 1.30–1.85 as middle, and > 1.85 as high incomes [15]. The categories of marital status were married or single. Participants who were married or living with partners were identified as “married,” while individuals who were separated, widowed, never married, or divorced were identified as “single.” Drinking and smoking statuses were categorized as “yes” or “no”. Hypertension was defined based on individuals diagnosed with hypertension, the usage of anti-hypertensive drugs, or if the mean systolic blood pressure was > 140 mmHg and/or mean diastolic blood pressure was > 90 mmHg. Diabetes was defined as diagnosed cases of diabetes or individuals using anti-diabetes drugs or insulin. The diagnosis of CVD was made based on congestive heart failure, self-reported heart attack, coronary heart disease, stroke, or angina. The individuals were allocated into four categories considering their BMI: underweight (BMI < 18.50 kg/m^2^), normal weight (BMI, 18.50–25.00 kg/m^2^), overweight (BMI, 25.00–30.00 kg/m^2^), and obese (BMI ≥ 30.00 kg/m^2^). The diagnosis of CKD was defined by the GFR and albuminuria categories based on the 2012 Kidney Disease Outcomes Quality Initiative guidelines [16]. The equations for computing the eGFR were in line with the Chronic Kidney Disease Epidemiology Collaboration.

### Statistical analysis

In this research, all analyses accounted for sampling weights. The new weights were computed by dividing the 2-year cycle weights by seven following the NHANES analysis guidelines. The DII scores were divided into tertiles from the lowest (T1) to the highest (T3). Continuous variables were expressed as means and standard error, whereas categorical variables were expressed as percentages or frequencies. Comparisons between the participants with and without hyperlipidemia were made by the student’s t-test for continuous variables and by the chi-squared test for categorical variables. Survey-weighted logistic regression analyses were carried out for evaluating the association between the prevalence of hyperlipidemia and DII after adjusting for the covariables. The potential nonlinear association between hyperlipidemia and DII based on Model 3 was evaluated using the restricted cubic spline (RCS). Model 3 was stratified by age, gender, drinking status, smoking status, diabetes, hypertension, CVD, and CKD to evaluate the association between DII and hyperlipidemia. R (v 4.2.2) was utilized for all the statistical analyses. *P-*values < 0.05 were determined as statistically significant.

## Results

### Characteristics of the study population

This study enrolled 70,190 participants from the NHANES from 2005 to 2018. After applying the exclusion and inclusion criteria, statistical analysis was carried out for 12,440 individuals. Table 1 illustrates the demographic and other characteristics of the participants. The individuals were classified into the non-hyperlipidemia and hyperlipidemia groups (categories) based on the absence and presence of hyperlipidemia, respectively. The prevalence of hyperlipidemia among the participants was 72.20%. The prevalence of hyperlipemia peaked in individuals aged [45, 65) years and declined in [65,) years. The hyperlipidemia prevalence differed significantly by race, marital status, education level, and poverty. Moreover, the rate of smoking and drinking was greater in individuals with hyperlipidemia than in non-hyperlipidemia individuals. The prevalence of hyperlipidemia differed significantly among the participants with diabetes, hypertension, CVD, and CKD. The Hb, glucose, and eGFR values varied considerably between the non-hyperlipidemia and hyperlipidemia categories. Notably, individuals with hyperlipidemia generally had higher DII scores than those without hyperlipidemia (Table 1).

**Table 1:** Demographic and other features of participants from the NHANES 2005–2018

	**Total (*N* = 12,440)**	**Non-hyperlipidemia (*N* = 3458)**	**Hyperlipidemia (*N* = 8982)**	**P**
**Age**				** < 0.01**
[18,45)	5127(41.21)	2151(67.04)	2976(36.59)	
[45,65)	4379(35.20)	853(24.22)	3526(41.97)	
[65,)	2934(23.59)	454(8.74)	2480(21.44)	
**Gender**				0.15
Male	6236(50.13)	1764(51.06)	4472(49.01)	
Female	6204(49.87)	1694(48.94)	4510(50.99)	
**Race**				** < 0.01**
White	5704(45.85)	1428(65.74)	4276(71.61)	
Black	2426(19.50)	830(13.34)	1596(9.27)	
Mexican	1929(15.51)	506(8.19)	1423(7.85)	
Other	2381(19.14)	694(12.73)	1687(11.27)	
**Marital status**				** < 0.01**
Single	4861(39.08)	1512(40.84)	3349(33.35)	
Married	7579(60.92)	1946(59.16)	5633(66.65)	
**Education**				** < 0.01**
Less than high school	2836(22.8)	659(12.49)	2177(15.97)	
High school	2866(23.04)	726(20.93)	2140(24.15)	
College	6738(54.16)	2073(66.58)	4665(59.88)	
**Poverty**				0.07
Low	3733(30.01)	1054(21.74)	2679(19.61)	
Middle	1661(13.35)	435(10.29)	1226(10.66)	
High	7046(56.64)	1969(67.97)	5077(69.73)	
**Drinking status**				0.58
No	1636(13.15)	458(10.63)	1178(10.23)	
Yes	10,804(86.85)	3000(89.37)	7804(89.77)	
**Smoking status**				** < 0.01**
No	6811(54.75)	2079(58.92)	4732(52.87)	
Yes	5629(45.25)	1379(41.08)	4250(47.13)	
**Diabetes**				** < 0.01**
No	10,733(86.28)	3262(96.46)	7471(87.55)	
Yes	1707(13.72)	196( 3.54)	1511(12.45)	
**Hypertension**				** < 0.01**
No	7140(57.40)	2539(77.93)	4623(56.04)	
Yes	5300(42.60)	919(22.07)	4359(43.96)	
**CVD**				** < 0.01**
No	11,070(88.99)	3314(97.27)	7756(88.60)	
Yes	1370(11.01)	144( 2.73)	1226(11.40)	
**CKD**				** < 0.01**
No	10,272(82.57)	3085(91.56)	7187(84.54)	
Yes	2168(17.43)	373( 8.44)	1795(15.46)	
**BMI**				** < 0.01**
Normal	3382(27.19)	1409(43.75)	1973(21.97)	
Underweight	187(1.50)	114(3.30)	73(0.81)	
Overweight	4127(33.18)	1041(30.10)	3086(34.36)	
Obesity	4744(38.14)	894(25.85)	3850(42.86)	
**eGFR (ml/min/1.73m**^**2**^**)**	95.07(0.36)	102.77(0.56)	91.88(0.35)	** < 0.01**
**Hb (g/dl)**	14.39(0.03)	14.31(0.04)	14.43(0.03)	**0.01**
**Glucose (mmol/L)**	5.88(0.02)	5.48(0.02)	6.05(0.03)	** < 0.01**
**DII**	1.42(0.03)	1.28(0.05)	1.48(0.04)	** < 0.01**
**DIIT**				** < 0.01**
T1	4146(33.33)	1254(38.79)	2892(34.28)	
T2	4152(33.38)	1124(32.46)	3028(34.34)	
T3	4142(33.29)	1080(28.75)	3062(31.38)	

^1^Data are presented as Mean ± SE or n (%)

^2^*P* value was calculated by Student’s t test and chi-square test

^3^DII tertile ranges: Tertile 1 = -4.68 to 0.80; Tertile 2 = 0.80 to 2.62; Tertile 3 = 2.62 to 5.50

### Association between the prevalence of hyperlipidemia and DII

Table 2 depicts the findings of the multivariable logistic regression analysis for identifying the independent risk factors for hyperlipidemia. To further examine the relationship between hyperlipidemia and DII, the DII scores were divided into tertiles as follows: T1: DII score, − 4.68 to 0.80; T2: DII score, 0.80 to 2.62; and T3: DII score, 2.62 to 5.50. When the DII was considered a continuous variable, it was considerably linked to hyperlipidemia in the crude model (odds ratio [OR] = 1.06, 95% confidence interval [CI] = 1.03–1.09; *P* < 0.01). The association remained statistically significant following adjustment for the underlying confounding variables in Models 1, 2, and 3.

**Table 2:** Logistic regression analysis of the association between the prevalence of hyperlipidemia and DII

	**Crude**		**Model**^**1**^		**Model**^**2**^		**Model**^**3**^	
	**95%CI**	**P**	**95%CI**	**P**	**95%CI**	**P**	**95%CI**	**P**
**DII**	1.06(1.03,1.09)	** < 0.01**	1.05(1.02,1.08)	** < 0.01**	1.05(1.02,1.08)	** < 0.01**	1.05(1.02,1.08)	** < 0.01**
**DIIT**								
T1								
T2	1.20(1.05,1.36)	**0.01**	1.17(1.01,1.35)	**0.03**	1.16(1.01,1.34)	**0.04**	1.15(1.00,1.33)	0.06
T3	1.24(1.09,1.40)	** < 0.01**	1.20(1.04,1.38)	**0.01**	1.17(1.02,1.35)	**0.03**	1.18(1.03,1.37)	**0.02**
**P for trend**		** < 0.01**		**0.01**		**0.02**		**0.02**
**Age**								
[18,45)								
[45,65)			3.00(2.57,3.51)	** < 0.01**	2.57(2.18,3.03)	** < 0.01**	2.57(2.18,3.04)	** < 0.01**
[65,)			4.28(3.59,5.09)	** < 0.01**	2.92(2.40,3.55)	** < 0.01**	3.02(2.47,3.69)	** < 0.01**
**Gender**								
Male								
Female		0.14	1.09(0.97,1.23)		1.15(1.02,1.30)	**0.03**	1.47 (1.26,1.71)	** < 0.01**
**Race**								
White								
Black			0.60(0.51,0.70)	** < 0.01**	0.58(0.49,0.68)	** < 0.01**	0.71(0.60,0.85)	** < 0.01**
Mexican			0.92(0.78,1.10)	0.37	0.98(0.82,1.17)	0.82	1.00(0.84,1.19)	1.00
Other			1.06(0.90,1.26)	0.47	1.07(0.90,1.28)	0.44	1.12(0.94,1.33)	0.22
**Marital status**								
Single								
Married			1.20(1.05,1.36)	**0.01**	1.20(1.05,1.36)	**0.01**	1.21(1.06,1.38)	** < 0.01**
**Education**								
Less than high school								
High school			0.89(0.74,1.07)	0.21	0.91(0.75,1.10)	0.32	0.90(0.75,1.09)	0.28
Collage			0.74(0.64,0.85)	** < 0.01**	0.78(0.67,0.90)	** < 0.01**	0.78(0.67,0.90)	** < 0.01**
**Poverty**								
Low								
Middle			1.04(0.86,1.25)	0.73	1.05(0.86,1.28)	0.61	1.05(0.86,1.28)	0.63
High			1.01(0.89,1.16)	0.89	1.06(0.92,1.22)	0.43	1.05(0.91,1.21)	0.48
**BMI**								
Normal								
Underweight			0.47(0.33,0.66)	** < 0.01**	0.46(0.32,0.66)	** < 0.01**	0.45(0.32,0.65)	** < 0.01**
Overweight			2.18(1.91,2.50)	** < 0.01**	2.12(1.85,2.42)	** < 0.01**	2.09(1.82,2.40)	** < 0.01**
Obesity			3.49(2.97,4.11)	** < 0.01**	3.08(2.62,3.63)	** < 0.01**	3.04(2.59,3.58)	** < 0.01**
**Drinking status**					1.13(0.96,1.33)	**0.15**	1.13(0.96,1.33)	0.15
**Smoking status**					1.10(0.98,1.24)	0.11	1.08(0.96,1.22)	0.22
**Diabetes**					1.64(1.30,2.09)	** < 0.01**	1.71(1.35,2.17)	** < 0.01**
**Hypertension**					1.36(1.20,1.55)	** < 0.01**	1.36(1.19,1.55)	** < 0.01**
**CVD**					2.15(1.63,2.83)	** < 0.01**	2.23(1.69,2.95)	** < 0.01**
**CKD**					1.05(0.87,1.26)	0.63	1.06(0.88,1.29)	0.51
**Hb (g/dl)**							1.15(1.10,1.21)	** < 0.01**

^1^adjusted for age, gender, race, marital status, education, poverty, BMI

^2^adjusted for age, gender, race, marital status, education, poverty, BMI, drinking status, smoke status, Diabetes, Hypertension, CVD, CKD

^3^adjusted for age, gender, race, marital status, education, poverty, BMI, drinking status, smoke status, Diabetes, Hypertension, CVD, CKD, Hb

When DII was considered a categorical variable, the analysis in the crude model showed that the participants in T2 and T3 had a greater prevalence of hyperlipidemia in contrast with T1 as a reference. The associations remained statistically significant in Models 1, 2, and 3. Following adjustment for age, marital status, educational status, race, poverty, and BMI, the relationship between higher DII scores and prevalence of hyperlipidemia remained significant (T2: OR = 1.17, 95% CI = 1.01–1.35; T3: OR = 1.20, 95% CI 1.04–1.38). In Model 2, the OR (95% CI) of hyperlipidemia for T2 and T3 was 1.16 (1.01–1.34) and 1.17 (1.02–1.35), respectively. In Model 3, the OR (95% CI) of hyperlipidemia for T2 and T3 was 1.15 (1.00–1.33) and 1.18 (1.02–1.37), respectively. Additionally, age, female gender, black race, married status, college education, BMI, diabetes, hypertension, CVD and Hb level were considerably linked to the prevalence of hyperlipidemia in Model 3.

**Fig. 2:**
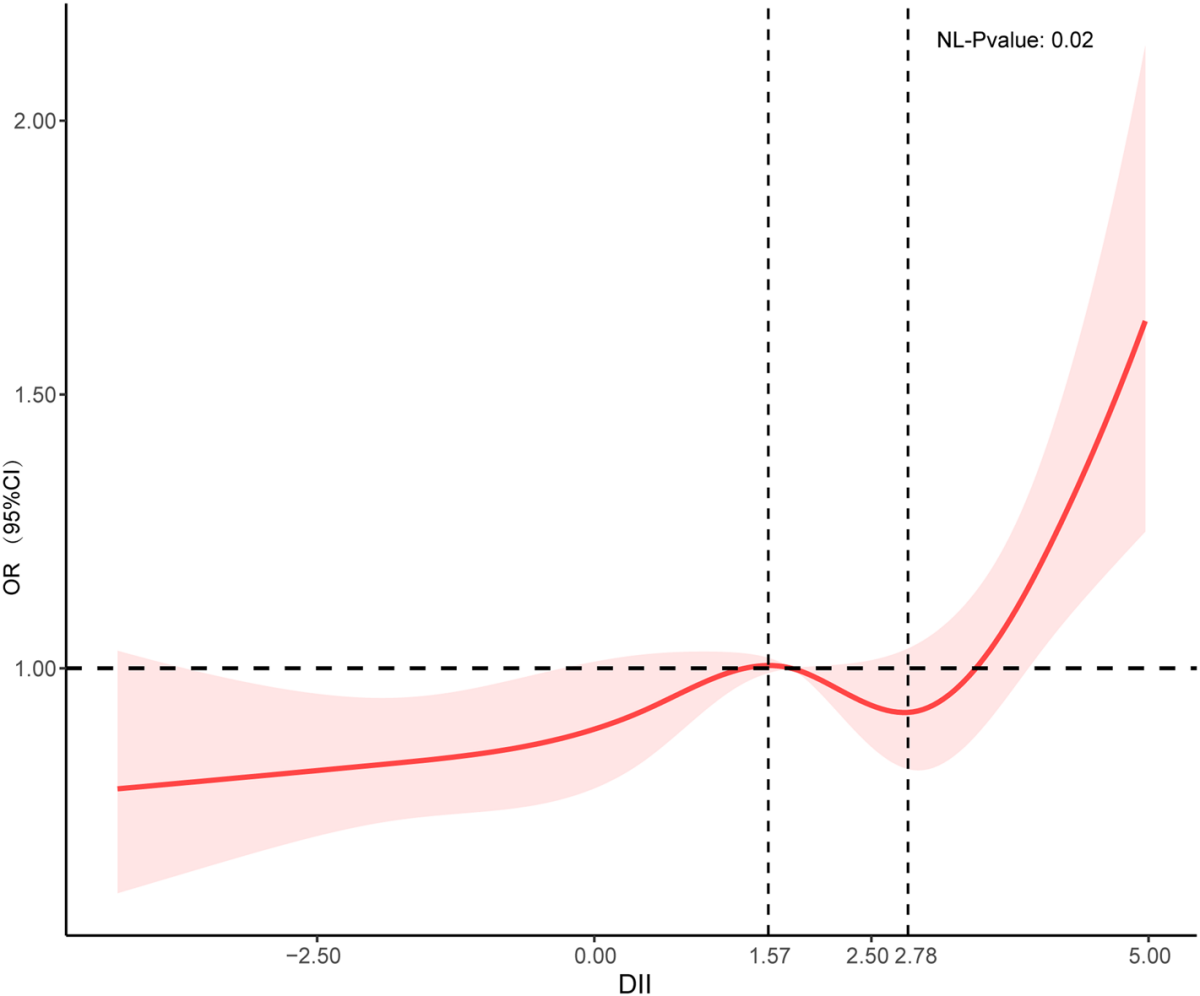
RCS depicts the association between DII and hyperlipidemia The red line represents OR, and the red transparent area represents 95% CI. ORs results are adjusted based on Model 3. (OR, odds ratio; CI, confidence interval)

RCS was performed to model the nonlinear effect of DII on the prevalence of hyperlipidemia. As shown in Fig. 2, the curve was divided into three sections by two inflection points which were 1.57 and 2.78. When the DII was less than 1.57, the OR was increased but the maximum value was < 1. The pro-inflammatory diets (0 < DII < 1.57) were protective and the protective effect diminished. When the DII was greater than 1.57 and less than 2.78, the pro-inflammatory diets were also protective. The OR was tended to decrease until the DII score reached approximately 2.78. The protective effect increased progressively with increasing DII. When the DII was greater than 2.78, the OR increased sharply (Fig. 2). The protective effect waned and gradually turned into a risk factor.

### Stratification analysis

**Fig. 3:**
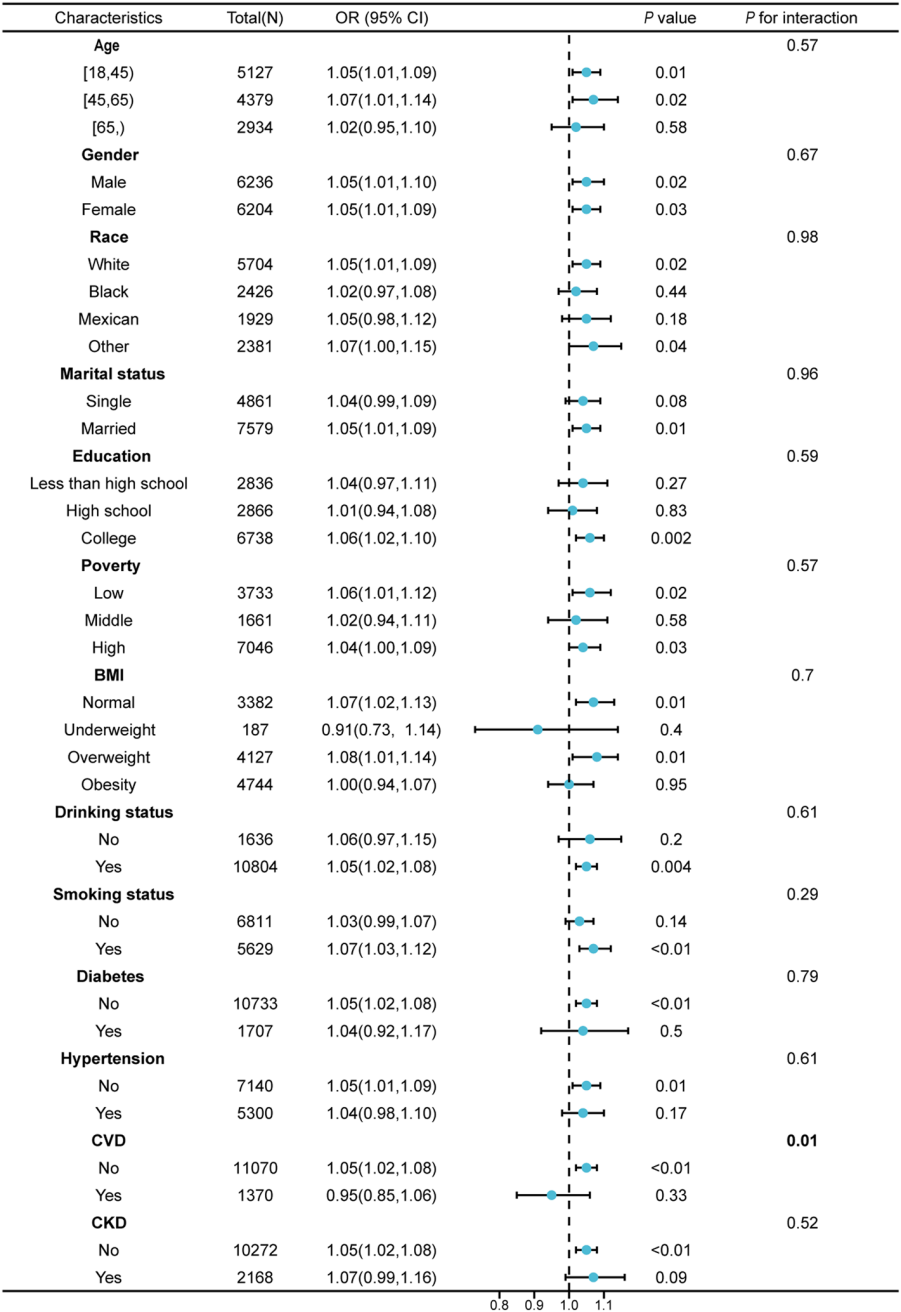
Subgroup analysis of the relationship between DII and hyperlipidemia. The outcome is adjusted for all covariables, with the exception of the corresponding stratification variable, glucose and eGFR

The analysis was carried out by dividing the participants into subgroups based on age, gender, race, BMI, marital status, education, smoking status, hypertension, CVD, and CKD. In the statistical model, the DII was considered a continuous variable. As illustrated in Fig. 3, the relationship between DII and hyperlipidemia remained consistent in all subgroups except for CVD subgroup. The association between DII and hyperlipidemia was more obvious in participants without CVD (OR = 1.05, 95% CI = 1.02–1.06) in contrast with individuals with CVD (OR = 0.95, 95% CI = 0.85–1.06) (Fig. 3). *P* for interaction was < 0.05 in the CVD subgroup.

## Discussion

Hyperlipidemia has a high prevalence in the US, and 72.20% of the participants in this study were diagnosed with hyperlipidemia. Hyperlipidemia is the primary underlying cause of CVD, which poses a heavy burden on the healthcare system [17, 18]. This cross-sectional survey analyzed the link between the prevalence of hyperlipidemia and DII in adult participants, and it was found that individuals with hyperlipidemia had greater DII scores. The prevalence of hyperlipidemia showed a remarkable association with DII, even after accounting for relevant confounders. Thus, DII serves as an independent risk factor for hyperlipidemia. Elucidating the relationship between DII and hyperlipidemia will facilitate timely interventions by adjusting dietary patterns. Furthermore, reducing the prevalence of hyperlipidemia will contribute to reduce the prevalence of its complications.

The DII is a new analytic tool designed to evaluate the inflammatory potential of diets and is often used to predict chronic disease-related endpoints. This is the first research conducted to report the association between hyperlipidemia and the inflammatory potential of diets calculated utilizing DII. The RCS visualized the association between DII and hyperlipidemia. When the DII was below 2.78, the level of inflammation caused by the pro-inflammatory dietary was insufficient to increase the prevalence of hyperlipidemia. However, when the DII was greater than 2.78, the level of inflammation caused by the pro-inflammatory dietary increased, and DII gradually turned into a risk factor for hyperlipidemia. The results of stratification analysis showed that there was an interaction between CVD and DII, indicating that the effect of DII on hyperlipidemia was different between CVD and non-CVD group. CVD and hyperlipidemia are closely related. CVD patients may intent to pay more attention to health management, especially diet, which may modify and weak the effect of DII on hyperlipidemia. The relatively stronger associations of the DII and hyperlipidemia were observed in the non-CVD participants. Highly inflammatory diets increased the prevalence of hyperlipidemia in participants of non-CVD. In other words, the participants of non-CVD with high DII score may benefit the most from diet interventions.

The biologic mechanisms underlying the association between DII and Hyperlipidemia remain unclear. Nonetheless, previous studies have provided insights into the probable mechanisms. The pro-inflammatory diet may increase levels of inflammatory cytokines by affecting immune mechanisms and oxidative stress [19]. Hyperlipidemia is primarily caused by an imbalance in lipid metabolism, which is strongly related to inflammatory processes [20]. In the past decade, the effects of diet on the immune system have been observed. Diet plays a role in the regulation of inflammation, and inflammation could alter various lipid metabolisms [21, 22]. Different nutrients in the diet modulate the pro-inflammatory and anti-inflammatory pathways [23]. The Western diet, which is high in saturated fatty acids and cholesterol, has pro-inflammatory properties [24]. Pro-inflammatory diets activate the innate sensor cells-recognizing microbe-associated molecular patterns receptor, and this activation can trigger pro-inflammatory cytokines production, including interleukin (IL)-6 and C-reactive protein (CRP) [25–27]. Several studies have shown that DII is associated with circulating concentrations of CRP, IL-6 and tumor necrosis factor-α [28–30]. In contrast, the Mediterranean diet, which is rich in unsaturated fatty acids, has anti-inflammatory properties [31]. The MUFA and PUFA play a significant role in immunological responses by suppressing the genes related to inflammation [32]. DII was significantly positively correlated with oxidative stress indicators including serum bilirubin, iron, and albumin [33]. These findings suggest that higher DII may increase oxidative stress, activate inflammatory response and induce lipid metabolism disorder. These mechanisms may underlie the association between DII and Hyperlipidemia, warranting further exploration.

### Comparisons with other studies and the contribution of this study to the existing knowledge

Most of the previous studies focused on exploring the relationship between diet patterns and lipid levels. This study quantified the dietary inflammatory intakes. The link between the prevalence of hyperlipidemia and the inflammatory potential of a diet was determined. Notably, a pro-inflammatory diet does not necessarily lead to a greater prevalence of hyperlipidemia. The prevalence of hyperlipidemia is elevated by the pro-inflammatory diet only in cases when the DII score is > 2.78.

### Strengths and limitations

This research is the first to investigate the relationship between DII and hyperlipidemia and find the threshold DII score that affects hyperlipidemia. However, this research has certain limitations. Firstly, as in any cross-sectional study design, unmeasured confounders may have been missed. Secondly, DII calculation using the 24-h dietary recall methods may lead to recall bias. Third, this study was conducted for the US population; hence, demography, economy, and geography may have affected the results. Further studies including a wider geographic population and more accurate diet data are warranted to validate the findings.

## Conclusion

This research offers a scientific basis for dietary management among individuals with hyperlipidemia. Evaluating the DII of individuals with hyperlipidemia could help to adjust the diet structure, balance nutrition, and improve the quality of life. Furthermore, this research offers an important reference for the early prevention of hyperlipidemia, especially for individuals with a DII score > 2.78, suggesting that they should consume more anti-inflammatory nutrients to reduce the risk of hyperlipidemia.

### Data availability

The datasets used and analyzed during the current study are available from the corresponding author on reasonable request.

## Data Availability

The data of the study are available from the corresponding author on reasonable request.

## Abbreviations

BMI: Body mass index
CKD: Chronic kidney disease
CRP: C-reactive protein
CVD: Cardiovascular disease
DII: Dietary inflammatory index
eGFR: Estimated glomerular filtration rate
Hb: Hemoglobin
IL: Interleukin
MUFA: Monounsaturated fatty acids
NHANES: National Health and Nutrition Examination Survey
OR: Odd ratio
PUFA: Polyunsaturated fatty acid
RCS: Restricted cubic spline
