# Correction: A cross‑sectional study exploring the relationship between the dietary inflammatory index and hyperlipidemia based on the National Health and Nutrition Examination Survey (2005–2018)

**DOI:** 10.1186/s12944-023-01924-x

**Published:** 2023-09-27

**Authors:** Yu Han, Xijuan Jiang, Yabin Qin, Yile Zhao, Guying Zhang, Chao Liu

**Affiliations:** 1 Department of Pharmacy, Hebei Children’s Hospital, Shijiazhuang, Hebei People’s Republic of China; 2 Department of Laboratory Animal Science, Hebei Medical University, Shijiazhuang, Hebei People’s Republic of China; 3 Hebei Key Lab of Laboratory Animal Science, Shijiazhuang, Hebei People’s Republic of China; 4 Department of Pharmacology, Hebei Medical University, Shijiazhuang, Hebei People’s Republic of China

**Correction: Lipids in Health and Disease 22, 140 (2023)**

10.1186/s12944-023-01908-x

Following publication of the original article [1], the authors requested to update Table 2.

The corrections are as follows:

1. In Table 2, the covariate of “Sex” should be “Gender”.
2. In Table 2, in the covariate of education, “Less” should be “Less than high school”, “High” should be “High school”.

The original article [1] has been updated.
